# The use of mechanistic reasoning in assessing coronavirus interventions

**DOI:** 10.1111/jep.13438

**Published:** 2020-07-15

**Authors:** Jeffrey K. Aronson, Daniel Auker‐Howlett, Virginia Ghiara, Michael P. Kelly, Jon Williamson

**Affiliations:** ^1^ Centre for Evidence‐Based Medicine Nuffield Department of Primary Care Health Sciences Oxford UK; ^2^ Department of Philosophy and Centre for Reasoning, School of European Culture and Languages University of Kent Canterbury UK; ^3^ Primary Care Unit, Department of Public Health and Primary Care, Institute of Public Health University of Cambridge Cambridge UK

**Keywords:** coronavirus, evidence‐based medicine, mechanisms, mechanistic reasoning

## Abstract

**Rationale:**

Evidence‐based medicine (EBM), the dominant approach to assessing the effectiveness of clinical and public health interventions, focuses on the results of association studies. EBM+ is a development of EBM that systematically considers mechanistic studies alongside association studies.

**Aims and objectives:**

To explore examples of the importance of mechanistic evidence to coronavirus research.

**Methods:**

We have reviewed the mechanistic evidence in four major areas that are relevant to the management of COVID‐19.

**Results and conclusions:**

(a) Assessment of combination therapy for MERS highlights the need for systematic assessment of mechanistic evidence. (b) That hypertension is a risk factor for severe disease in the case of SARS‐CoV‐2 suggests that altering hypertension treatment might alleviate disease, but the mechanisms are complex, and it is essential to consider and evaluate multiple mechanistic hypotheses. (c) Confidence that public health interventions will be effective requires a detailed assessment of social and psychological components of the mechanisms of their action, in addition to mechanisms of disease. (d) In particular, if vaccination programmes are to be effective, they must be carefully tailored to the social context; again, mechanistic evidence is crucial. We conclude that coronavirus research is best situated within the EBM+ evaluation framework.

## 1 INTRODUCTION

Evidence‐based medicine (EBM) provides the dominant approach to assessing the effectiveness of clinical and some public health interventions. In order to assess whether an intervention *A* results in outcome *B*, EBM relies heavily on what we shall call “association studies,” which measure *A* and *B* to assess whether they are probabilistically dependent, conditional on potential confounders, which are often measured at the same time. Not all association studies are equal, for example, experimental studies such as randomized controlled trials (RCTs) are favoured over observational studies, ceteris paribus. Studies other than association studies tend to be regarded as less useful by EBM. For example, mechanistic reasoning, which appeals to features of the mechanisms by which the intervention is hypothesized to lead to the outcome and to the mechanistic studies that investigate these features, is viewed as inferior to association studies by present‐day EBM.^1^

**FIGURE 1:**
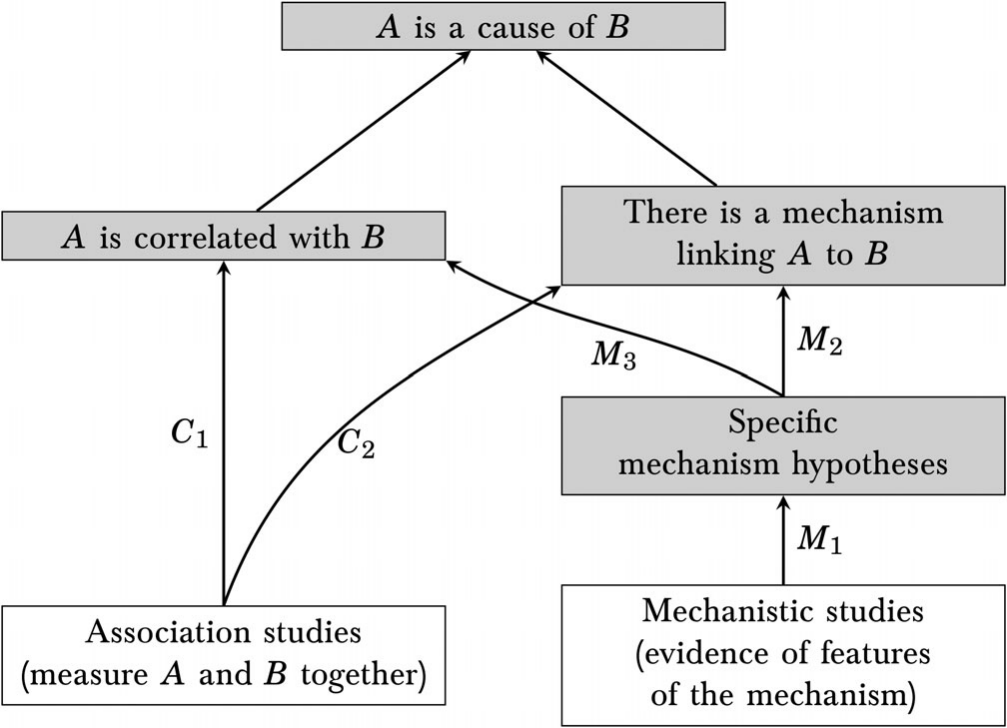
Evidential relationships for establishing a causal claim.^3^ The existence of an appropriate correlation and an appropriate mechanism together confirm causation—it is not enough to have one without the other. Arrows signify potential positive or negative evidential relationships

EBM+ is a development of EBM that treats mechanistic studies on a par with association studies.^2^ Figure 1 portrays the EBM+ view of the assessment of a causal claim. Association studies provide direct evidence that the putative cause *A* and the putative effect *B* are correlated (pathway *C*_1_ in Figure 1). But correlation is insufficient for causation: a correlation may be attributable to chance, bias, uncontrolled confounders, inappropriately controlled colliders, or relationships other than causation. What distinguishes correlations that are causal from those that are spurious is the existence of a mechanism complex, by which instances of *A* explain instances of *B*. So, in order to establish causation, one needs to establish the existence of a mechanism (or mechanisms) of action as well as the existence of a correlation. Experimental studies such as RCTs are valuable precisely because they can indirectly support the existence of a mechanism (channel *C*_2_), by making confounding and bias less likely to corrupt the relationship between the dependent and independent variables. But mechanistic studies also provide evidence of the existence of a mechanism, by confirming or disconfirming specific hypotheses about features of the mechanism complex linking *A* and *B* (channels *M*_1_ and *M*_2_). (In some cases, these features can also support or undermine the claim that *A* and *B* are correlated—channel *M*_3_.) Reasoning that proceeds along channels *M*_1_, *M*_2_, and/or *M*_3_ is mechanistic reasoning. Mechanistic reasoning is particularly pertinent when specific hypotheses about key features of the mechanism complex are established (or ruled out) by mechanistic studies (see Section 3).

Given this more nuanced picture of causal assessment, it can be important to explicitly and systematically scrutinize mechanistic studies when assessing causal claims. This need is now often recognized when assessing the effects of environmental exposures^4^ but less so when assessing the effects of interventions^5^, ^6^ and of infectious diseases.^7^

In this paper, we aim to redress the balance by showing that there is a need to assess mechanistic studies explicitly and systematically when interrogating the effects of interventions in infections with coronaviruses. Indeed, this need is particularly urgent in diseases such as SARS, MERS, and COVID‐19 (due respectively to SARS‐CoV‐1, MERS‐CoV, and SARS‐CoV‐2), because outbreaks are rapid, limiting the opportunity to conduct high‐quality RCTs. Thus, association studies on their own tend to provide evidence that is too weak to establish causation. When rapid outbreaks are coupled with severe disease, effective interventions need to be identified very quickly. In these circumstances, only by considering mechanistic studies alongside association studies is it possible to build an evidence base that distinguishes those interventions that are effective from those that are ineffective.

In the following sections, we provide several examples of the importance of mechanistic evidence to coronavirus research. The assessment of combination therapy for MERS highlights the difficulties that can be encountered when suggesting treatments on the basis of poor mechanistic reasoning (Section 2). That treatment for hypertension is a risk factor for severe disease in the case of SARS‐CoV‐2 suggests that altering hypertension treatment might alleviate disease, but the mechanisms are complex, and it is essential to consider and evaluate more than one mechanistic hypothesis (Section 3). To successfully limit the spread of an infection, public health interventions need to take account of all relevant social mechanisms, rather than just targeting risk factors. Moreover, interventions shown to be effective in one country cannot be successfully extrapolated to other countries unless the relevant social mechanisms are sufficiently similar, and this also calls for scrutiny of mechanistic evidence (Section 4). Finally, if vaccination programmes are to be effective, they must be carefully tailored to the social context; again, mechanistic evidence is crucial (Section 5). We conclude that coronavirus research is best situated within the EBM+ evaluation framework (Section 6).

## 2 COMBINATION THERAPY FOR MERS

One strategy in seeking interventions for diseases caused by novel viruses is to repurpose existing drugs. This strategy makes much use of evidence from mechanistic studies: evidence that the drug has some action against the novel virus in the laboratory, both in vitro and in vivo in experimental animals, is used to justify the decision to use the treatment clinically. COVID‐19 is no different. For example, the antimalarial drug hydroxychloroquine inhibits SARS‐CoV‐2 replication in vitro,^8^ leading to the suggestion that it would be a good intervention for COVID‐19. The motivation for this strategy is twofold. Because of the novelty of the virus, there are no disease‐specific drugs ready for testing. And as a result of the intensity of the disease, there is a pragmatic motivation for using compounds that may not yet have been rigorously tested. The end goal in the standard approach is still testing of compounds in randomized trials. However, high‐quality trials are time‐consuming. Responses to COVID‐19 thus rely heavily on the use of evidence from mechanistic studies.

Can this strategy be improved by taking an EBM+ approach? Standard EBM says little about the role mechanistic reasoning can play in attempts to identify effective treatments. As a result, mistakes may be made in how such evidence is handled. On the other hand, EBM+ takes mechanistic studies into account and offers explicit guidance regarding their evaluation. The following analysis of the way repurposing was carried out in response to MERS shows how the EBM+ approach uses mechanistic reasoning to identify effectiveness.

As a repurposing strategy, a *combination* of an interferon (IFN) and ribavirin has been suggested for treating MERS. The mechanism of action of IFN is fairly well known,^9^ and there was some evidence that replication of MERS‐CoV is inhibited by IFN in vitro and in vivo^10^, ^11^. A major problem for the use of IFN was that it only inhibited the virus at clinically unobtainable concentrations. However, a synergistic effect was observed by using it in combination with ribavirin, in vitro inhibition of MERS‐CoV being observed at clinically obtainable concentrations of both compounds.^12^, ^13^ Moreover, rhesus macaques infected with MERS‐CoV displayed signs of recovery when given combination therapy.^14^ At no point did these studies establish that this treatment would be effective, but this evidence nevertheless provided the motivation for clinical use of combination therapy.

The problem here is that merely having some evidence from mechanistic studies is not enough to conclude that a mechanism of action exists. Arguably, for a treatment to be put forward for clinical use, the existence of a mechanism linking the treatment and recovery from the disease must be very plausible. However, evidence that a drug inhibits viral replication in vitro is only weak evidence that it will inhibit viral replication in vivo. Evaluating the extent to which mechanistic studies confirm the plausibility of mechanistic hypotheses is something that EBM+ adds to standard EBM.

Demonstration of inhibition of viral replication in vitro and in vivo in animals is not enough to conclude that the mechanism is relevant to the clinical outcome in humans for the following reasons. In addition to viral replication, the pathology of MERS may include a mechanism by which the immune system contributes to lung damage, even after viral load is reduced.^15^ Reductions in lung pathology in rhesus macaques supported combination therapy; hence, one might be tempted to say that there is evidence for both important features of the mechanism by which combination therapy affects outcomes in MERS. The problem with this conclusion is that rhesus macaques are inappropriate animals for investigating whether an intervention can reduce respiratory pathology. This is because they only develop a mild form of MERS that lacks the kind of severe lung damage observed clinically.^16^ Full evaluation of this evidence thus shows that it is only moderately plausible that a mechanism exists linking combination therapy and recovery from MERS in humans.

Of course, it will be no surprise to proponents of EBM that evidence from mechanistic studies may struggle to prove the existence of a relevant mechanism. However, it is not enough merely to note that results are not easily extrapolated from cell cultures or animals to humans, and to apply this scepticism rigidly to all experimental systems. Common marmosets, for example, develop a form of MERS very similar to that in humans, and evidence obtained using these animals is much more relevant to humans than evidence from rhesus macaques.^16^, ^17^ Without explicitly evaluating mechanistic evidence, it is not possible to distinguish a plausible (strong) mechanistic hypothesis from an implausible (weak) hypothesis. Moreover, when repurposing drugs, compounds that are more likely to be effective should be prioritized. These considerations favour the EBM+ approach, as does an analysis of the evidence for another putative treatment for MERS.

Mycophenolic acid was proposed as an intervention for MERS, based on evidence that replication of MERS‐CoV was inhibited in vitro.^18^ Moreover, it was more efficacious than combination therapy.^19^, ^20^, ^21^ Accordingly, mycophenolic acid was suggested as a potential treatment for MERS and saw some clinical use.^22^ However, mycophenolic acid is an immunosuppressant, and its mechanism of action involves selective depletion of the DNA and RNA precursor guanosine in T cells.^23^ Indeed, it was subsequently found to be associated with greater mortality in common marmosets.^17^ Testing of mycophenolic acid should not have proceeded to clinical testing; the immunosuppression mechanism should have been considered. EBM+ requires that the whole *complex of mechanisms* by which mycophenolic acid operates should be scrutinized, rather than isolated mechanistic hypotheses. This practice is not always followed, and in this case, a compound made its way into clinical testing that should not have. This is thus another case in which a full evaluation of evidence from mechanistic studies would improve on the current repurposing strategy.

## 3 ANTI‐HYPERTENSIVE DRUGS AND THE INTENSITY OF COVID‐19

Pharmacological risk surveillance is another area in which an evidentially diverse approach to causal evaluation is desirable.^24^, ^25^ Knowing a mechanism that links a treatment and an adverse event can help explain their observed correlation. Take, for example, antihypertensive drugs that act on components of the renin‐angiotensin system (RAS) and might be either beneficial or harmful, as competing mechanistic hypotheses suggest.^26^, ^27^, ^28^

**FIGURE 2:**
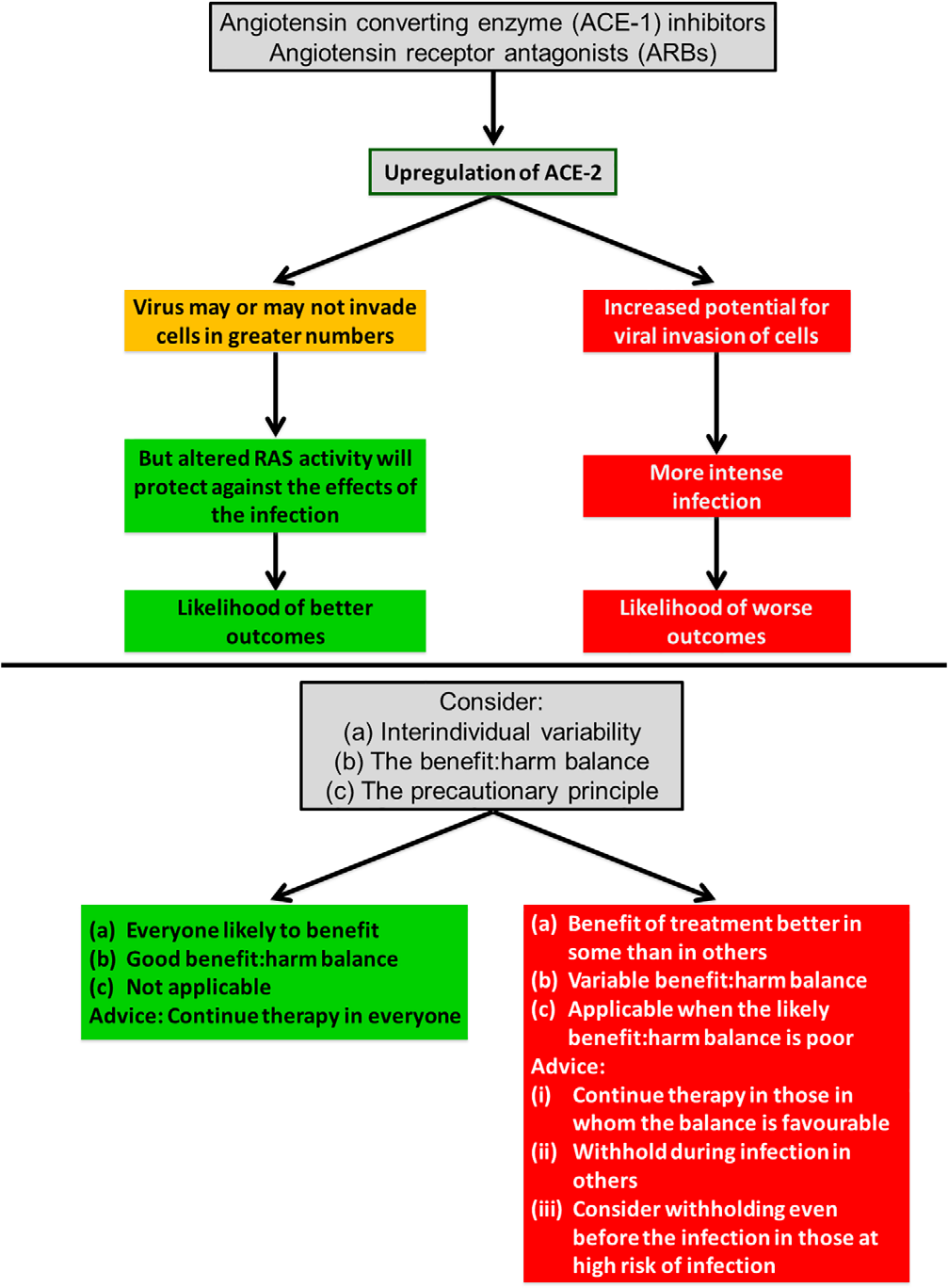
Two conflicting hypotheses about whether to recommend continuing treatment with antihypertensive drugs in the context of COVID‐19. The two hypotheses interpret increased activity of the renin‐angiotensin system (RAS) in different ways, leading to different pieces of advice regarding continuation of antihypertensive drug use (see Aronson and Ferner)^2^

Angiotensin converting enzyme 2 (ACE‐2) is the receptor to which SARS‐CoV‐2 binds to enter host cells.^29^, ^30^ Some antihypertensive drugs increase ACE‐2 expression. It has therefore been suggested that those drugs may worsen the intensity of COVID‐19 by providing a greater opportunity for SARS‐CoV‐2 to enter host cells.^27^ On the other hand, ACE‐2 protects against lung injury by regulating concentrations of angiotensin II, which is vasoconstrictive, pro‐inflammatory, and pro‐oxidative.^31^ Hence, it has been suggested that increased expression of ACE‐2 from using antihypertensive drugs might reduce the intensity of COVID‐19.^28^ This evidence suggests two mechanism hypotheses: H1, by which the drugs increase the intensity of the disease, and H2, by which they reduce it (Figure 2).

The status of H1 and H2 is problematic. There are two separate issues. First, the mechanistic studies from which the evidence is obtained have not been properly evaluated, and neither has the plausibility of either mechanism. The mechanism by which ACE2 protects against lung injury (H2) is fairly well established,^32^, ^33^ but the evidence of a protective effect against SARS‐CoV‐2 induced lung injury^34^ needs evaluation, since it may be insufficient to support the claim of a protective mechanism. For example, an error may have been committed during the study, or the experimental system and the target system may be dissimilar.^2^ Only by evaluating the evidence that supports the proposed mechanisms can this question be settled. Secondly, these mechanisms are plausibly not mutually exclusive. That they both operate is a distinct possibility, although if so, they probably operate in sequence rather than in parallel: by H1, more virions enter the host cell, and by H2, the damage caused by the virus is reduced. EBM+ recommends evaluating the whole complex of mechanisms at work. Focusing on isolated mechanisms risks missing interactions in the way they influence the intensity of the disease.

Despite the absence of high‐quality clinical trials, we nevertheless have some evidence of possible causal relationships. In this sort of scenario, the EBM+ approach enables evaluation of the plausibility of causality. We might obtain evidence of a correlation between antihypertensive drug therapy and the intensity of COVID‐19 through association studies with lower quality designs, in advance of better ones. Such studies cannot prove causation, because they are subject to biases. However, the EBM+ approach to causal evaluation combines this kind of evidence with (properly evaluated) evidence of mechanisms to obtain a better assessment of causality than either strand of evidence provides alone.^35^ While confounding, colliding, and biases remain problems for evidence of mere correlation between antihypertensive drugs and COVID‐19, if an established mechanism links the two variables, it may be possible to regard such biases as less plausible explanations of the observed association on COVID‐19 outcomes. This will particularly be the case when mechanistic reasoning is strong, and sensitivity analyses suggest that no individual bias is likely to have had a large effect. Moreover, the problem of predicting the overall effect of H1 and H2 (and any number of other mechanisms that may be operating on the same pathways) is resolved by establishing that either an injurious or protective effect is correlated with antihypertensive drugs, or that there is no correlation and so no overall effect. The reinforcement of both lines of evidence, a characteristic feature of EBM+, is thus more informative about how decisions about the use of antihypertensive drugs during COVID‐19 should be made. At the time of writing, the evidence of association between antihypertensive drugs and the intensity of COVID‐19 is inconclusive. While at first, studies showed an association between antihypertensives and reduced intensity, more recent studies have found no such association.^36^ But a judgement on the plausibility of causality is still better informed by evaluating and integrating both lines of evidence.

### 3.1 Strong mechanistic reasoning

The importance of mechanistic reasoning to conclusions about the effects of antihypertensive drugs depends on the extent to which mechanistic studies shed light on the relevant mechanisms. For mechanistic reasoning to be strong, key features of the mechanism complex should be well established, and this can be achieved by meeting the following conditions:

- Ideally, the operation of each feature should have been demonstrated in vitro in different types of relevant cells/tissues/organ, and in vivo in a range of species. When the experimental system differs from the target system (eg, a macaque model investigating a treatment in humans), a mechanism that exists in one experimental species and not another is unlikely to be helpful.
- Such demonstrations should include evidence that the mechanism exists, that enhancing it leads to measurable outcomes, and likewise for inhibiting it or abolishing it.
- Such demonstrations should involve different ways of doing this (eg, different agonists and antagonists, and genetic knock‐outs), and dose responsiveness should be demonstrated at concentrations that could plausibly occur in vivo.

Other criteria that, if fulfilled, would strengthen the mechanistic reasoning further include:

- Demonstrating the anatomical location of the mechanism and its relevance. Do strategic lesions cause predictable changes?
- Demonstrating the time course of the mechanism. Does it change in response to interventions such as upregulation or downregulation?
- Do genetic polymorphisms,^37^, ^38^, ^39^ physiological variables, diseases, and drug interactions alter it predictably?
- When any changes occur, is dose‐responsiveness maintained?

Establishing key features of the whole complex of relevant mechanisms through a variety of experimental sources thus makes mechanistic reasoning strong.

## 4 MECHANISMS BEYOND THE MICROBIOLOGICAL

Many public health interventions attempt to change or influence human behaviour. For decades, most of this effort has gone into preventing non‐communicable diseases—for example, by preventing smoking, obesity, and alcohol misuse and encouraging physical activity.^40^ Although human behaviour is also central to the transmission of infectious disease, efforts to change behaviour have mostly been applied to HIV, other sexually transmitted infections, and tuberculosis.

One important finding from work in non‐communicable diseases, as well as HIV, has been the realization that social and psychological mechanisms are relevant both to aetiology and to the effectiveness of preventive interventions.^41^, ^42^, ^43^ These include mechanisms linking a behaviour change intervention and a behavioural outcome, and how intervention content, mode of delivery, population, context, setting, exposure, engagement, and time and place all affect efficacy and effectiveness of interventions^44^, ^45^ (eg, https://www.humanbehaviourchange.org/).

A major obstacle to effective delivery of public health programmes has been confusion between etiological mechanisms and preventive mechanisms. The default is often to the assumption that if we know the cause, we know how to implement prevention.^41^ However, knowing that there is a very strong relationship between exposure to cigarette smoke and lung cancer tells you nothing about how to help someone stop smoking. The same applies to alcohol and liver disease or calorie consumption and obesity. Preventive mechanisms are not the same as etiological mechanisms. This has long been recognized in tobacco control, where preventive efforts have focussed on price, advertising, availability, opportunity, addiction, and peer pressure—on the mechanisms of behaviour rather than the aetiology of the disease. Mechanisms of prevention were also the focus of early HIV/AIDS intervention,^46^ but not for obesity, alcohol misuse, and physical activity, where most efforts still focus on explaining risk or advising caution, for example, recommending sensible alcohol consumption.

There are important lessons here for COVID‐19 in the United Kingdom. First, the behavioural mechanisms involved in the aetiology of the disease are the social vectors of transmission. They are complex. They determine vulnerability and risk, as well as the rates at which, and where, the disease will spread. These include mechanisms involving family structures and interaction patterns, occupational behaviour, urban density, housing occupation and overcrowding, workplace and retail environment structures and organization, as well as local social, economic, and cultural variation. However, there is nothing in the public record or the published models of the UK Government that suggests that these well‐known complexities were systematically built into UK models (https://www.gov.uk/government/groups/scientific‐advisory‐group‐for‐emergencies‐sage‐coronavirus‐covid‐19‐response). Furthermore, the predictive models were initially not only based on relatively small amounts of data but also on data from a very different cultural environment to the United Kingdom. The assumption that the cultures were similar enough to allow extrapolation is very tenuous. Above all, they were models of aetiology, not models of prevention, exemplifying the confusion mentioned above.

Secondly, the way the population engages with preventive measures is affected by the mechanisms we are describing. So acceptance of the measures introduced by the UK Government, such as closing schools, giving the police new powers, and shutting pubs, restaurants, and many workplaces (interventions that were based on the etiological models mentioned above), will be affected by trust in government and its messaging. Being able to act upon the specific UK Government messaging required, for example, having space to self‐isolate, reliable digital technology and internet access in the home, effective habitual coping skills, feelings of self‐efficacy, and resources such as credit cards or ready access to cash. Behavioural science has had a presence at the table,^47^ but there is little evidence in the actions and measures that have been put in place in the United Kingdom that the subtleties of human behaviour mechanisms have really been integrated into the thinking at policy level.

Thirdly, one needs to consider mechanisms that affect take‐up of interventions. The use of testing, for example, is based on the assumption that people will avail themselves of the test and understand what the result means. Not everyone will, and different individuals and groups will respond differently to the offer. This response is governed not only by availability, but also by the social and psychological mechanisms in play. The same will be true *inter alia* of other offers, such as tracing apps and any vaccine, should one become available.

These mechanisms are not as well understood as we need them to be, and there is an urgent need for programmes of research and evidence synthesis. These mechanisms will also have to be integrated into decision‐making processes in future coronavirus pandemics. The knowledge base, such as it is, is highly Anglo‐American‐centric, and its transferability to other settings is not known. The idea that strategies that have been used in North America and Europe could or should be applied elsewhere is not sound. Likewise, applying strategies that appear to have worked in South Korea, Singapore, or China to the United Kingdom are subject to the same uncertainty. The UK strategy of closing schools assumed that this protects children and families, because schools are potentially infection rich. However, this was premised on the biology of influenza, which thrives in schools, making children vectors of infection.^48^ But COVID‐19 is different. Children do not appear to be at great risk from SARS‐CoV‐2, though their infectivity towards adults is not clear at present. As a preventive mechanism, school closure is a blunt instrument. When schools reopen, various social and behavioural mechanisms come into play, such as parents' reluctance to return their children to school, teachers' fears of returning to work, and the need for transport to and from schools.

Applied in settings outside the United Kingdom, this strategy is blunter still. School closures have many potential consequences. These include, but are not limited to, increased risks from accidents and injury outside the relative safety of the school, sexual violence to young girls, pressures and demands on parents, including economic costs of staying away from work, living conditions in multiple‐generation households (which, unlike schools, are likely to be important cockpits of infection in COVID‐19), long‐term educational disadvantages through missing schooling (more likely to damage girls), and pressures on the social fabric to which schools contribute.^49^ All these will interact with local socio‐economic and cultural settings.

There are thus two strategies for jurisdictions that seek to establish public health measures to deal with coronavirus pandemics. One is to develop local solutions, building from the ground up, rather than naively importing solutions that may never have been understood properly in the countries that implemented them. The other is to extrapolate interventions that have worked well elsewhere. However, successful extrapolation requires a detailed evaluation of the similarities and differences between the relevant mechanisms in the source and target jurisdictions.^2^, ^50^ These two strategies are not mutually exclusive and can be combined.

## 5 TAILORING VACCINATION PROGRAMMES

By June 2020, researchers worldwide haddeveloped 149 vaccine candidates against COVID‐19, of which 17 are in clinicalevaluation and 132 in preclinical evaluation. Although the development of an effective vaccine will mark a major step forward, the mere existence of a vaccine will not ensure high uptake.

As noted in the “Tailoring Immunization Programmes” guide published by the WHO's Regional Office for Europe, mechanistic studies are crucial in order to understand the psychological, contextual, and social mechanisms that influence vaccination behaviours.^51^ In recent years, several vaccination studies have investigated barriers to vaccination, and have identified evidence, which confirms general mechanisms that appear to work in diverse situations, as well as more specific mechanisms that work only in particular contexts.^52^ Most of these mechanistic studies can provide useful insights for coronavirus vaccination and the assessment of its effects.

Some evidence, for instance, supports a behavioural mechanism known as the “intention‐behaviour gap,”^53^ which seems to work in various contexts. Sheeran and Webb^51^ observed that even though the intention to perform a behaviour and the behaviour itself are strongly correlated, manipulating the intention will not necessarily change behaviour. On the contrary, the authors argued that changes in intention generally do not immediately translate into behavioural changes. For instance, Smith et al^54^ reported that in the United Kingdom, at the end of the 2015 to 2016 vaccination campaign, over 70% of parents decided to vaccinate their children against seasonal flu, but only 53% progressed to vaccination. Sheeran and Webb^53^ identified several components of this “intention‐behaviour gap,” including forgetfulness, lapse in willpower, and procrastination, and this mechanistic discussion has helped policymakers to understand why some programmes did not work as predicted, and how to improve vaccination uptake.^52^

Not all of these mechanisms are equally important, nor do they function in the same way across contexts. Many studies have reported that the prevalence of some mechanisms that inhibit or promote vaccination behaviours differ between and within countries. For example, anti‐vaccine sentiment and its related behaviours appear to be more prevalent in high‐income than in low‐ and middle‐income countries.^55^

It follows that identification of the psychological, contextual, and social mechanisms that influence vaccine‐related behaviours is not only important in understanding why vaccination coverage is lower than expected, but is also crucial in developing new effective vaccination programmes. This consideration is even more relevant, when, as in the case of the COVID‐19 outbreak, it may not be possible to carry out large extended trials to test the effects of an intervention, and the limited correlational data available from impact evaluations cannot rule out the risk of bias. Systematically scrutinizing mechanistic studies can help policy makers to develop effective vaccination programmes in at least two different ways.

On the one hand, knowing that a mechanism operates in the target context helps policymakers avoid problems of external validity. For example, educational programmes aimed at increasing childhood vaccine uptake were reported to be effective in low‐income countries, where access to vaccination‐related information is difficult and parental knowledge is poor. However, similar programmes that focussed on parents with little knowledge about vaccines in high‐income countries did not show the same effect.^56^ Researchers argued that this difference was due not only to different levels of knowledge in the target populations, but also to different causes of educational barriers. While access to information is the main problem in low‐income countries, educational barriers to immunization in high‐income countries are often associated with beliefs about safety or importance, which in turn influence parents' decisions not to seek information.^57^, ^58^

**FIGURE 3:**
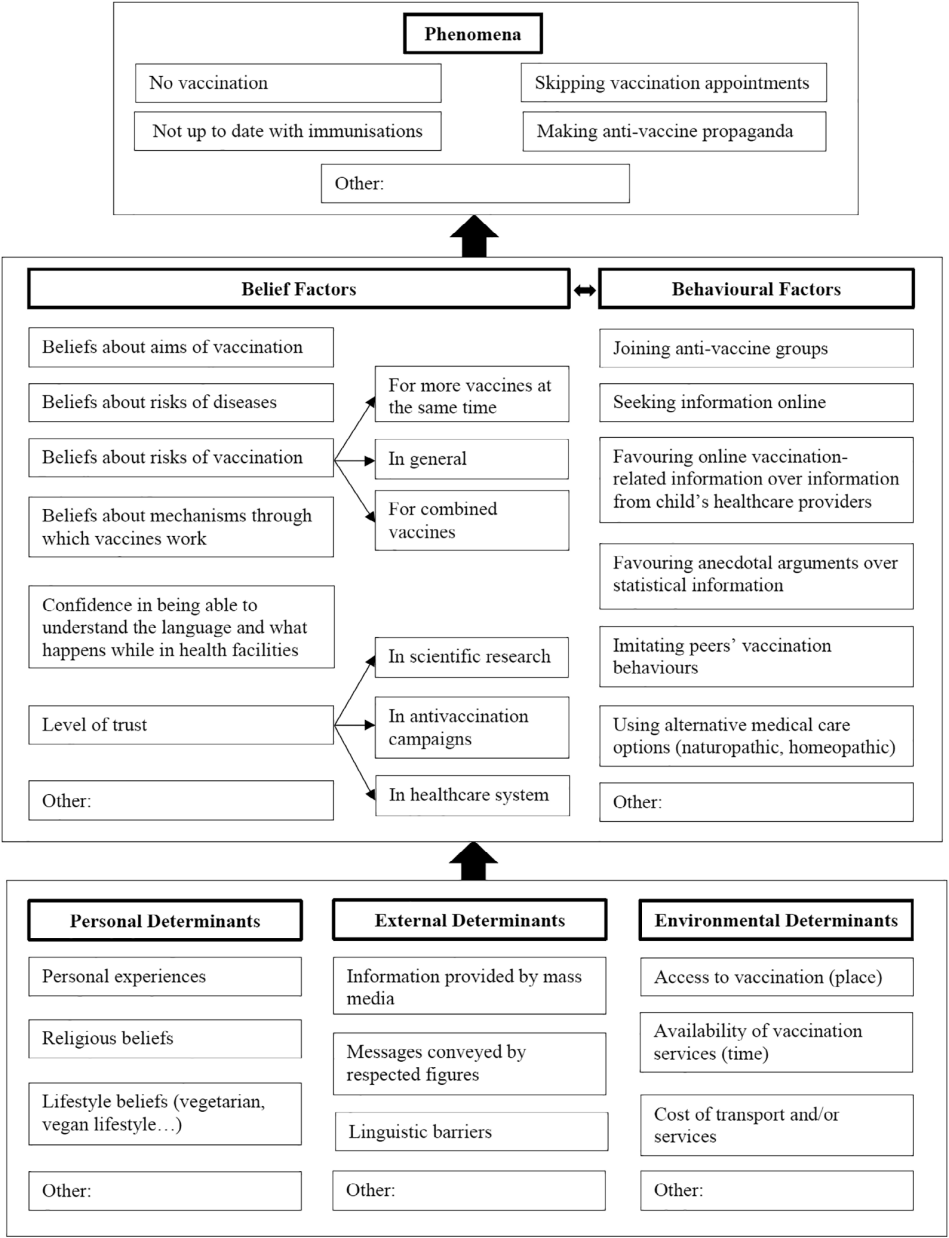
Factors that can influence vaccination status, motivated by EBM+ and the WHO's Tailoring Immunization Programmes.^49^ Personal, external, and environmental determinants influence belief and behavioural factors, which then bring about different phenomena relevant to vaccination status

On the other hand, identifying psychological, contextual, and social mechanisms can help policymakers to select the factors to target in vaccination programmes (Figure 3). For instance, the United Kingdom and other countries have recently emphasized the importance of herd immunity, and in these countries, it might appear sensible to develop programmes that promote vaccination by highlighting the altruistic nature of vaccination. There is, however, conflicting evidence about how a sense of altruism affects vaccine behaviours. While some studies have shown that understanding the benefits of herd immunity could increase vaccination uptake because people comprehend the altruistic nature of their behaviour,^59^, ^60^ others have shown that such knowledge can promote behaviours based on the view that as more people are vaccinated, individuals get less benefit from vaccination.^61^, ^62^ Awareness that the mechanisms that determine motivations for vaccination are complex has led some scientists to focus more on potential interventions to bridge the “intention‐behaviour gap.” Rather than aiming at influencing people's motivations for vaccination, such interventions focus on keeping intentions on people's minds and eliminating real or perceived barriers that might make taking action difficult for some individuals.^63^

In sum, the explicit and systematic assessment of mechanistic studies is essential for the development of effective vaccination programs in a given context.

## 6 SUMMARY AND RECOMMENDATIONS

We have presented a range of contexts in coronavirus research in which it is beneficial to systematically assess mechanistic studies alongside association studies. The cases we have discussed are illustrative rather than exhaustive. Indeed, we have not touched on assessment of medical devices, for which mechanistic reasoning is clearly essential; for example, the use of less invasive respiratory devices^64^ might be viewed as an early success of mechanistic reasoning in the COVID‐19 outbreak. (Although we have presented some considerations that bear on *how* mechanistic studies should be assessed, we refer the reader to Parkkinen et al,^2^ who provide a general account of EBM+ assessment, for more detail).

In the course of a coronavirus outbreak, it is essential to give neither too little weight nor too much weight to mechanistic reasoning. On the one hand, demanding high‐quality association studies, which usually do not exist in the early stages of an outbreak of a novel virus, can lead to inaction and many lives lost—lives that could be saved by considering the evidence base as a whole, which might warrant the use of particular interventions. On the other hand, advocating interventions solely on the basis of mechanistic reasoning that is of low quality can lead to a proliferation of ineffective interventions or ineffective extrapolations of interventions that are effective elsewhere. Thus, it is vital that mechanistic studies and mechanistic hypotheses are explicitly and systematically evaluated alongside association studies.

The relevant mechanism complexes typically involve social and psychological pathways, in addition to biomedical pathways. Social and psychological factors are key to uptake of and adherence to an intervention. Moreover, social behaviours can change radically during an epidemic, partly in response to perceptions about the organism and its effects and partly in response to public health interventions. Only by explicitly articulating and systematically assessing these potential mechanisms can one ensure that coronavirus interventions are effective.

## CONFLICT OF INTEREST

The authors declare no conflict of interest.
